# Microdose GnRH Agonist Flare-Up versus Ultrashort GnRH Agonist Combined with Fixed GnRH Antagonist in Poor Responders of Assisted Reproductive Techniques Cycles

**Published:** 2013-03-03

**Authors:** Maryam Eftekhar, Farnaz Mohammadian, Fariba Yousefnejad, Parisa Khani

**Affiliations:** 1 Department of Obstetrics and Gynecology, Research and Clinical Center for Infertility, Shahid Sadoughi University of Medical Sciences, Yazd, Iran; 2 Department of Obstetrics and Gynecology, Zanjan University of Medical Sciences, Zanjan, Iran; 3 Research and Clinical Center for Infertility, Shahid Sadoughi University of Medical Sciences, Yazd, Iran

**Keywords:** GnRH Agonist, GnRH Antagonist, Poor Responder, Assisted Reproductive Technology

## Abstract

**Background:**

This study compares the microdose flare-up protocol to the ultrashort gonadotropinreleasing hormone (GnRH) agonist flare combined with the fixed multidose GnRH antagonist protocol in poor responders undergoing ovarian stimulation.

**Materials and Methods:**

In this randomized clinical trial, 120 women who were candidates for assisted reproductive techniques (ART) and had histories of one or more failed *in vitro* fertilization (IVF) cycles with three or fewer retrieved oocytes were prospectively randomized into two groups. Group I (60 patients) received the microdose flare-up regimen and group II (60 patients) received the ultrashort GnRH agonist combined with fixed GnRH antagonist.

**Results:**

There were no significant differences between the groups in the number of used gonadotropin ampoules (p=0.591), duration of stimulation (p=0.610), number of retrieved oocytes (p=0.802), fertilization rate (p=0.456), and the number of transferred embryos (p=0.954). The clinical pregnancy rates were statistically similar in group I (10%) compared with group II (13.3%, p=0.389).

**Conclusion:**

According to our results, there is no significant difference between these protocols for improving the ART outcome in poor responders. Additional prospective, randomized studies with more patients is necessary to determine the best protocol (Registration Number: IRCT201105096420N1).

## Introduction

Despite considerable advancements over the past decade in assisted reproduction, poor responders remain an important challenge. These patients have more problems in fertilization, embryo quality, and pregnancy. Poor response to ovarian stimulation occurs in 9-18% of assisted reproductive technique (ART) cycles. However there is no specific definition for poor responders, thus a comparison of outcomes from various protocols is challenging (1-3). The most common definition of a poor responder is based on increased basal FSH, an inadequate ovarian response, low oestradiol (E2) levels to ovarian stimulation by FSH/HMG, and lower number of retrieved oocytes (3-6).

Several strategies are available to improve ovarian stimulation outcome in poor responders, including increase the dose of the gonatropin that is being used and administration of gonadotropinreleasing hormone (GnRH) analogs (agonists or antagonists). The use of clomiphene citrate, aromatase inhibitors, growth hormones, transdermal testosterone, corticosteroids, estradiol or aspirin are recommended as adjuvant therapies (4, 7-10).

One of the most successful protocols for ovarian stimulation of poor responders is the microdose flareup protocol (11-13). The basic hypothesis of this approach involves administration of a minimal dose of GnRH-a to stimulate gonadotropin release and minimize premature ovulation (14). GnRH antagonists represent an alternative in the management of poor responders (15). Antagonists act to rapidly block gonadotropin receptors so ovarian stimulation can be initiated before administration of the GnRH antagonist. As a result these agents prevent a premature LH surge but do not suppress early follicular development (16-18). GnRH antagonists have no flair effect on follicular development compare with GnRH agonists.

Our hypothesis is to compare the microdose Gn- RH-a flare-up protocol with the combined stimulatory effect of GnRH agonists and immediate suppression of the GnRH antagonist in a unique protocol that may be a valuable new strategy for ovarian stimulation of poor responders, causing an improved ART outcome. In this study we compare the microdose flare-up protocol to the ultrashort GnRH agonist flare combined with the fixed multidose GnRH antagonist protocol in poor responders undergoing ART cycles.

## Materials and Methods

### Patients

**Fig 1:**
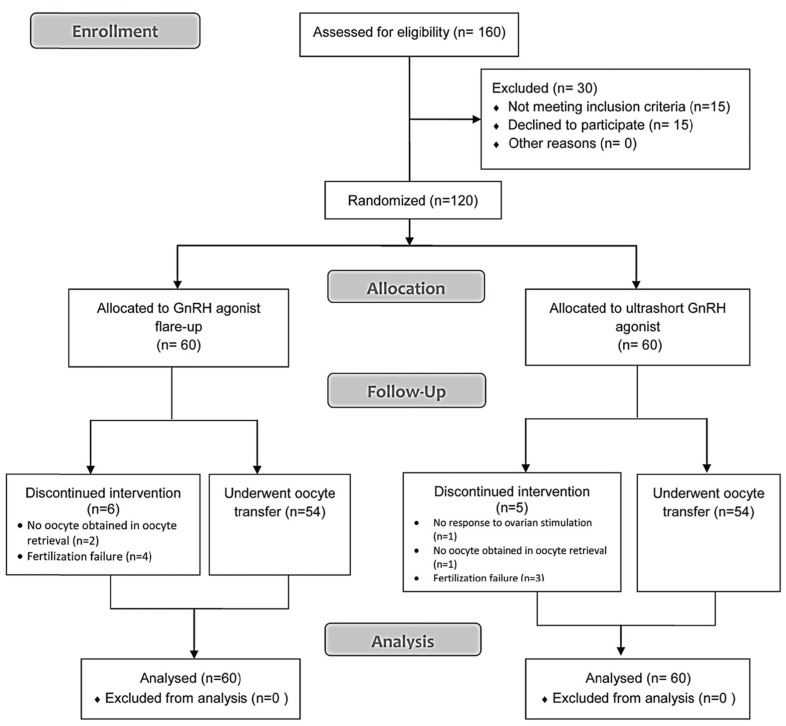
Study flowchart

A total of 120 poor responder women who referred to the Yazd Fertility and Infertility Center of Shahid Sadoughi University of Medical Sciences from June 2007 to July 2009 were enrolled in this randomized clinical trial. This randomized, controlled study was approved by the Ethics Committee of Yazd Fertility and Infertility Center and was undertaken in accordance with CONSORT guidelines (Fig 1). All patients signed a written consent form before initiation of the treatment cycles.

All included patients had a history of one or more failed IVF cycles with three or less retrieved oocytes. There was no age limitation for participants. We excluded patients with: 1. body mass index (BMI) >30, 2. endocrine or metabolic disorders, 3. history of endometriosis or 4.severe male factor (azspermia).

Patients were randomly allocated into two groups by the use of sealed envelopes. In group I (60 patients) the microdose flare-up regimen was used. Group II (60 patients) were treated with the ultrashort GnRH agonist combined with fixed GnRH antagonist regimens.

### Ovarian stimulation protocols

All patients received oral contraceptive pills during their previous menstrual cycle. In group I patients received 0.05 mg subcutaneous buserelin (Suprefact, Serono) injections twice daily from the first day of the cycle that continued until the day of the HCG injection. Ovarian stimulation was started from the third day of the patient,s menstrual cycle by intramuscular (IM) injections of HMG (Menogon, Ferring, Germany) at a dose of 300 IU per day. Follicular monitoring began from the ninth day of the cycle by serial vaginal ultrasonography and measurement of serum E2 levels. I.M. injections of 10000 IU HCG (Pregnyl; NV Organon, Oss, The Netherlands) were injected when at least 2 follicles ≥18 mm were observed on ultrasonography.

Group II patients received buserelin (Suprefact, Serono), 0.5 mg/ subcutaneous (SC) per day from the first day of the menstrual cycle, which was continued for three consecutive days. HMG (Menogon, Ferring) at 300 IU per day was started on day three of the cycle. The GnRH antagonist (Cetrorelix, Serono Laboratories, Aubonne, Switzerland) at a dose of 0.25 mg SC per day was started when the dominant follicle size reached a diameter of 14 mm. Follicular monitoring by vaginal ultrasonography and estradiol level measurement began on the ninth day of the cycle. Patients received 10000 IU HCG (Pregnyle, NV Organon, Oss, The Netherlands) when at least 2 follicles that were ≥17-18 mm in diameter were observed by ultrasonography. In both groups oocyte retrieval was performed 34-36 hours after the HCG injection, using a 17 gauge needle under vaginal ultrasonography guidance. Conventional IVF or intracytoplasmic sperm injection (ICSI) was appropriately performed. Fertilization rate was defined as the ratio of number of oocytes with pronuclei observed at least 18 hours after IVF or ICSI to the number of retrieved oocytes. A Labotect catheter (Labotect, Gottingen Germany) was used to transfer the embryos at 48-72 hours following oocyte retrieval. Luteal phase support began with I.M. injections of progesterone in oil (progesterone, Aburaihan Co., Tehran, Iran) at a dose of 100 mg daily on the day of oocyte retrieval and continued until documentation of fetal heart activity by ultrasound. Primary outcome was the clinical pregnancy rate per cycle.

Clinical pregnancy was identified as observation of fetal heart activity by transvaginal ultrasonography performed three weeks after a positive β-hCGv (β-hCG >50 IU/L) two weeks after embryo transfer. This means that the ultrasonography was actually 5 weeks after embryo transfer.

### Statistical analysis

The Statistical Package for Social Sciences (SPSS, version 15.0 for Windows; SPSS Inc., Chicago, IL) was used for data analysis. Student’s ttest and chi-square test were used to detect significant difference (p<0.05) between the variables. All data were expressed as mean ± SD.

## Results

We randomly recruited 120 patients, with 60 patients in each treatment group. There were no significant differences in mean female age, basal FSH and duration of infertility between both groups (Table 1). After randomization, 6 patients in group I did not have embryo transfers. Of these, 2 patients had no oocytes in oocyte retrieval and 4 had fertilization failure. In group II, 5 patients did not have embryo transfers, of which 1 patient had no response to ovarian stimulation, 1 patient had no oocytes obtained during oocyte retrieval, and 3 patients had fertilization failure. All 11 patients were part of the final analysis and not excluded from the study. There were no patients lost to follow up. Table 2 shows the cycle characteristics and ART outcomes.

**Table 1:** Patient’s characteristic in two groups (Mean ± SD)


	Microdose flare-up	GnRH agonist/antagonist	P value

**Female age (Y)**	33.31 ± 6.02	35.33 ± 4.11	0.139
**Infertility duration (Y)**	9.91 ± 5.10	8.00 ± 5.32	0.310
**Basal FSH (mIU/ml)**	9.43 ± 2.11	9.91 ± 1.90	0.315


**Table 2:** ART outcome in two groups (Mean ± SD)


	P value	GnRH agonist/ antagonist	Microdose flare-up

**No. of used gonadotropin ampoules**	0.591	44.12 ± 8.20	45.20 ± 6.93
**Duration of stimulation (Days)**	0.610	11.60 ± 1.32	11.42 ± 1.61
**No. of retrieved oocytes**	0.802	4.61± 3.53	4.42 ± 3.63
**No. of transferred embryos**	0.954	2.44 ± 2.10	2.31 ± 2.41
**Fertilization rate (%) (Per cycle)**	0.458	62 ± 27	58 ± 30
**Clinical pregnancy rate (%) (Per cycle)**	0.389	13.3%	10%


There were no significant differences between groups in the number of used gonadotropin ampoules, the duration of stimulation, the number of retrieved oocytes, fertilization rate and the number of transferred embryos. The clinical pregnancy rate (per cycle) was 10% (6) in group I and 13,3% (8) in group II, which was statistically similar in both groups (p=0.389), although there was a trend toward a higher clinical pregnancy rate in the ultrashort agonist/ antagonist protocol.

## Discussion

The best stimulation protocol for poor responders remains controversial. An adequate stimulation protocol should lead to an acceptable rate of cancellation, retrieve an adequate number of oocytes, obtain good quality embryos, and eventually achieve maximum pregnancy and live birth rates (20). Several stimulation regimens have been proposed for poor responders. Some have improved the ovarian response to stimulation but none were able to significantly improve the pregnancy rate (21). The most common protocols for management of poor responders are the microdose flare-up protocol and antagonist protocol. The microdose flare-up protocol benefits from the release of endogenous gonadotropin in the early follicular phase of the cycle through administration of a low dose GnRH agonist to enhance response to ovarian stimulation. However this approach may lead to a premature LH surge and compromise the cycle, which in turn can affect oocyte and embryo quality, in addition to synchronization between the embryo and endometrium (19). Addition of gonadotropin to an ovarian stimulation protocol prevents premature LH surge without suppression of early follicular development (22).

In the present study, we compared the microdose GnRH agonist flare-up and ultrashort GnRH agonist that was combined with the fixed multidose GnRH antagonist. According to our findings, poor responders demonstrated similar outcomes. The number of used gonadotropin ampoules, duration of simulation, and the number of retrieved oocytes were statistically similar in both groups. Fertilization and pregnancy rates per cycles were similar in both groups. Antagonist consumption in a poor responder stimulation protocol is associated with the possibility of decreasing the number of gonadotropin ampoules used and reducing the duration of stimulation. However Scott and Navot have studied the microdose GnRH flare-up protocol for low responder women in an ART protocol and reported a lower cancellation rate, increased number of retrieved oocytes, and higher pregnancy rates in these patients (23). A number of previous studies have evaluated the effect of a GnRH antagonist in the management of poor responders and determined that these protocols improved implantation and pregnancy rates (24, 25). The first study that has compared an agonist-antagonist protocol with the microdose flare-up protocol was reported by Berger et al. (26). They showed that addition of antagonist to an agonist in the ovarian stimulation protocol was associated with reduced gonadotropin consumption and duration of stimulation. However, as with our study, they have demonstrated that the agonist-antagonist is not inferior to the microdose flare-up protocol in poor responders (26). Erden et al. in a pilot study demonstrated that the agonistantagonist protocol compared to microdose flareup was associated with higher peak estradiol levels, more mature and fertilized oocytes, and higher clinical pregnancies (27).

Orvieto et al. compared an ultrashort GnRH agonist combined with a flexible multidose GnRH antagonist and microdose flare-up administration of GnRH. In contrast to our results, they found higher numbers of mature oocytes and embryos in the ultrashort GnRH agonist/ antagonist group. Pregnancy rate was also significantly higher in this group (28). In contrast to our study, they used a flexible multidose GnRH antagonist and defined poor responder as the retrieval of fewer than five oocytes in the previous ART cycle.

The successful end-point of ART is to obtain a live, healthy infant (17). Studies have shown similar ART outcomes or only increased numbers of retrieved oocytes and/or obtained embryos. They could not recommend a unique protocol for increasing live births in poor responders (21, 29, 30).

## Conclusion

Although our findings showed no statistically difference in clinical pregnancy rate and ART outcome between these two protocols, however this new protocol could possibly be considered as a future ovarian stimulation protocol for poor responders. Additional, large randomized prospective studies are recommended to further evaluate the role of agonist-antagonist in poor responder protocols.
